# The increasing impact of length of stay “outliers” on length of stay at an urban academic hospital

**DOI:** 10.1186/s12913-021-06972-6

**Published:** 2021-09-09

**Authors:** Andrew H. Hughes, David Horrocks, Curtis Leung, Melissa B. Richardson, Ann M. Sheehy, Charles F. S. Locke

**Affiliations:** 1 Department of Medicine, Johns Hopkins University School of Medicine, Baltimore, MD USA; 2 Department of Care Coordination/Clinical Resource Management, Johns Hopkins Hospital, 600 N Wolfe St / Blalock 823, Baltimore, MD 21287 USA; 3 Department of Medicine, Division of Hospital Medicine, University of Wisconsin School of Medicine and Public Health, Madison, WI USA

**Keywords:** Length of stay, Hospital resource utilization, Outlier length of stay

## Abstract

**Background:**

As healthcare systems strive for efficiency, hospital “length of stay outliers” have the potential to significantly impact a hospital’s overall utilization. There is a tendency to exclude such “outlier” stays in local quality improvement and data reporting due to their assumed rare occurrence and disproportionate ability to skew mean and other summary data. This study sought to assess the influence of length of stay (LOS) outliers on inpatient length of stay and hospital capacity over a 5-year period at a large urban academic medical center.

**Methods:**

From January 2014 through December 2019, 169,645 consecutive inpatient cases were analyzed and assigned an expected LOS based on national academic center benchmarks. Cases in the top 1% of national sample LOS by diagnosis were flagged as length of stay outliers.

**Results:**

From 2014 to 2019, mean outlier LOS increased (40.98 to 45.11 days), as did inpatient LOS with outliers excluded (5.63 to 6.19 days). Outlier cases increased both in number (from 297 to 412) and as a percent of total discharges (0.98 to 1.56%), and outlier patient days increased from 6.7 to 9.8% of total inpatient plus observation days over the study period.

**Conclusions:**

Outlier cases utilize a disproportionate and increasing share of hospital resources and available beds. The current tendency to exclude such outlier stays in data reporting due to assumed rare occurrence may need to be revisited. Outlier stays require distinct and targeted interventions to appropriately reduce length of stay to both improve patient care and maintain hospital capacity.

## Background

In an increasingly competitive and resource limited healthcare marketplace, appropriate and efficient utilization management by hospitals is of growing import and scrutiny [1]*.* One metric of hospital utilization and quality of care is length of stay (LOS) [2]*.* The arithmetic and geometric mean length of stay by hospital diagnosis is provided each year by the Centers for Medicare & Medicaid Services (CMS) to allow for benchmarking and calculating certain Medicare payments [3]. Greater LOS is associated with increased adverse events and lower care quality [4–7]*.* Additionally, a significant proportion of total hospital days may lack medical necessity or be associated a preventable delay in hospital discharge [8–11].

The majority of inpatient hospital stays insured by traditional Medicare and other government payers in the United States are paid under a diagnosis-related group (DRG) system. This confers a lump-sum payment for most of the care provided during an inpatient hospitalization. The formula and computation of payment under a DRG system is complex, but it is partially based on the mean of total costs of inpatient stay for a particular diagnosis [12]*.* One important factor in inpatient stay cost is the length of stay of the hospitalization [13]*.* Because DRG payments are fixed based on diagnoses, co-morbidities, and procedures performed, hospitals are generally incentivized under the DRG system to shorten LOS. However, the Medicare DRG payment system recognizes that the disproportionate costs of a few markedly high-cost hospitalizations would not be fairly compensated under the DRG model [14]. CMS annually publishes the threshold criteria for a case to qualify as a “cost outlier”. These cases are paid under a separate calculation based on actual resource use [14].

In monitoring inpatient care quality and efficiency, one metric of interest to hospitals is LOS by DRG and how this compares to peer institutions. Vizient Inc. provides a source of such benchmarking, collecting and analyzing individual case-level data for both community and academic medical center (AMC) hospitals [15, 16]. The AMC “peer group” includes approximately 175 U.S. academic medical centers. These peer groups allow analysis of LOS performance by diagnosis, adjusted for severity of illness and other factors for inter-hospital comparison. These data can additionally be analyzed with and without “length of stay outliers”. Length of stay outliers are defined by Vizient as those cases in which the LOS is >99th percentile for a given DRG. Such outlier cases have a disproportionate impact on arithmetic mean LOS, and as a result are often excluded from operational analyses of LOS. However, a recent study of a VA hospital in Washington State found that only 3.4% of all hospitalizations in the study cohort represented 27% of total bed days of care [17]. Furthermore, many such long stay patients have barriers to discharge beyond the medical need for acute care hospitalization, such as inability to identify appropriate post-discharge placement, which would potentially compound their influence on effective utilization of hospital resources [17].

The Johns Hopkins Hospital (JHH) is a 1003-bed academic quaternary medical center located in an urban setting in Baltimore, MD. We sought to quantify the effect of LOS outliers on length of stay at our hospital and whether any “outlier effect” was increasing or decreasing over the past several years. We hypothesized if outliers were increasing, they would be using an increasingly disproportionate share of hospital resources and additional attention may be needed to address the specific needs of this population.

## Methods

To assist in hospital management and administration, JHH contracts with Vizient Inc., a data analytics company that aggregates and groups U.S. hospital data to provide insights and benchmarking. All inpatient records, including data elements such as ICD-10 codes, procedure codes, and demographic information, are sent to Vizient for processing. Inpatient hospitalizations are assigned an expected length of stay (ELOS) and identified as either length of stay “outliers” or “non-outliers”. These data are then shared with JHH and re-linked with patient records for analysis. Johns Hopkins Hospital is a member of the Vizient AMC hospital peer group.

Inpatient hospitalizations are grouped by Vizient and assigned to one of 393 Vizient LOS regression model groups referencing AMC data. These model groups are based on the Medicare Severity-Diagnosis Related Group (MS-DRG) system and can include a single or multiple similar MS-DRGs. These groups are derived from procedure and ICD-10 coding of individual records. Once hospitalizations are assigned to a DRG group, multivariate regression analysis is used to assign each hospitalization an ELOS. The explanatory variables included in the models are based on diagnoses, procedures, complications and comorbidities, demographics, admit and discharge source/status, and primary payer/socioeconomic status. Vizient collects all-payer data from the 175 U.S. AMCs to create these regression models, and they are updated yearly. Hospitalizations with lengths of stay that comprise the top 1% of observed LOS for each model group are classified as LOS outliers. Given that the top 1% of each group includes the hospitalizations for all 175 AMCs, individual hospitals may have more or less than 1% of its discharges in each model group be classified as outliers. Correspondingly, individual hospitals may have more or less than 1% of total discharges be classified as outliers. Outpatient observation hospital days includes hospitalizations spent entirely under outpatient observation status, as well as days spent in observation as a part of hospitalizations that were subsequently “converted” to inpatient.

For the time period of January 1, 2014 to December 31, 2019, JHH discharged 170,185 medicine, surgery and neurology inpatient hospitalizations. Oncology, pediatric and psychiatry hospitalizations were excluded from the analysis. 540 inpatient discharges (0.3%) were not assigned an ELOS or outlier status and were excluded. Typically, an ELOS was not assigned due to a discharge being retroactively reclassified from observation to inpatient status, or rare cases where a hospitalization bill was flagged as discharge/not billed or voided by finance. 169,645 cases were assigned an ELOS and outlier status by Vizient and were used in further analysis.

LOS observed/expected (LOS O/E) is calculated as observed (actual) length of stay days divided by the model’s expected length of stay days assigned for each discharge. LOS O/E index is calculated for all inpatient hospitalizations as the sum of total observed days divided by the sum of the model’s total expected days. A LOS O/E index of 1.00 indicates a hospital’s observed LOS is on par with the case mix-adjusted mean of the 175 AMCs in the Vizient academic peer group. This index can be calculated including or excluding LOS outliers. Outlier days are the sum of inpatient days for all discharges that are classified as LOS outliers. Outlier days as a percent of total days are calculated as the percentage of total inpatient + observation days that are attributable to LOS outliers.

## Results

### LOS O/E

**Fig. 1:**
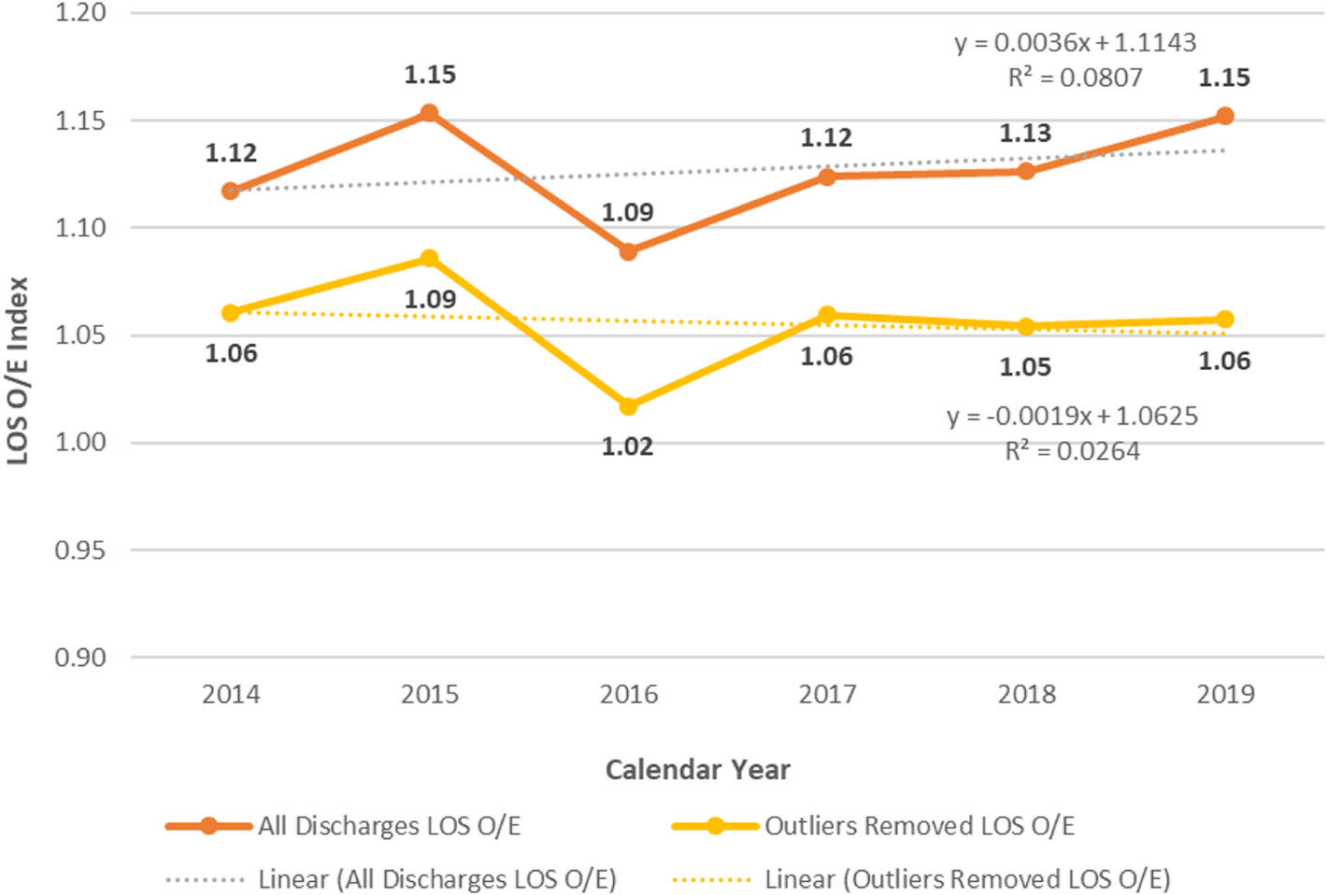
LOS O/E Index Trends (Inpatient Hospitalizations)

For the calendar years 2014 to 2019 the total discharges LOS O/E for inpatient admissions on the medicine, surgery and neurology services remained fairly constant, ranging from 1.09 to 1.15 (Fig. 1). The LOS O/E with outliers removed also remained relatively constant, though at a lower O/E, ranging from 1.02 to 1.09 (Fig. 1).

### Mean LOS

**Fig. 2:**
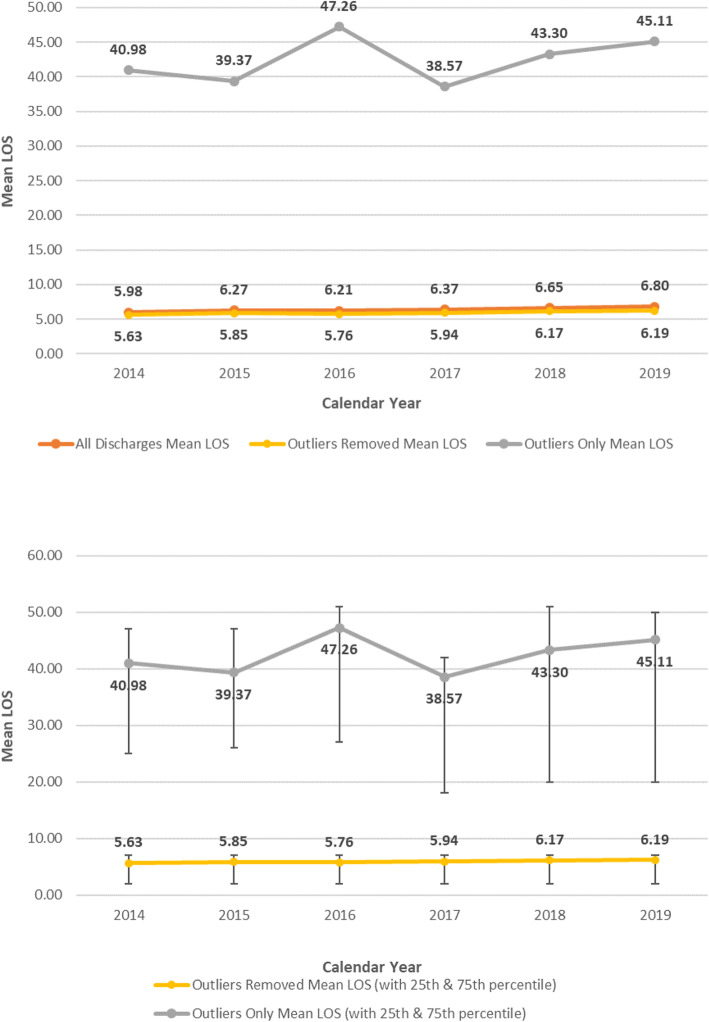
Observed Mean LOS (Inpatient Hospitalizations). a. Observed Mean LOS (Inpatient Hospitalizations) with 25th and 75th percentile

During the same period there was an overall increase in the observed mean LOS for inpatient hospitalizations. Evaluating all cases (outliers + non-outliers), the mean LOS increased from 5.98 days in 2014 to 6.80 days in 2019 (Fig. 2). Similarly, the mean LOS increased from 5.63 days in 2014 to 6.19 days in 2019 when excluding outliers, though the 25th and 75th percentile remained static over the study period at 2 and 7 days (Fig. 2). The mean LOS for outliers also increased during the study period, from 40.98 days in 2014 to 45.11 in 2019 (Fig. 2). The 25th percentiles for outlier only cases LOS varied from 18 days in 2017 to 27 days in 2016 (Fig. 2). The 75th percentiles for outlier only cases LOS varied from 42 days in 2017 to 51 days in 2016 (Fig. 2).

### Inpatient discharges

**Fig. 3:**
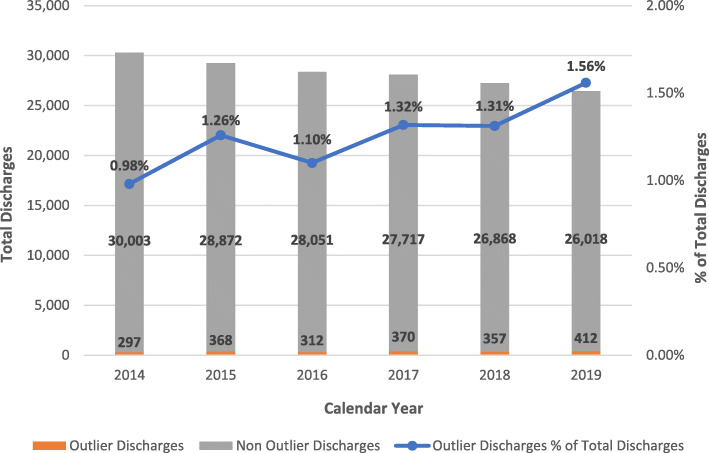
Total Inpatient Discharges, Outlier Discharges Count and % of Total

The total annual discharges trended down from 30,300 in 2014 to 26,430 in 2019 (Fig. 3). Conversely, the total number of annual discharges for outliers alone increased from 297 in 2014 to 412 in 2019 (Fig. 3). Additionally, outlier discharges as a percent of total inpatient discharges increased from 0.98% in 2014 to 1.56% in 2019 (Fig. 3).

### Outpatient days

**Fig. 4:**
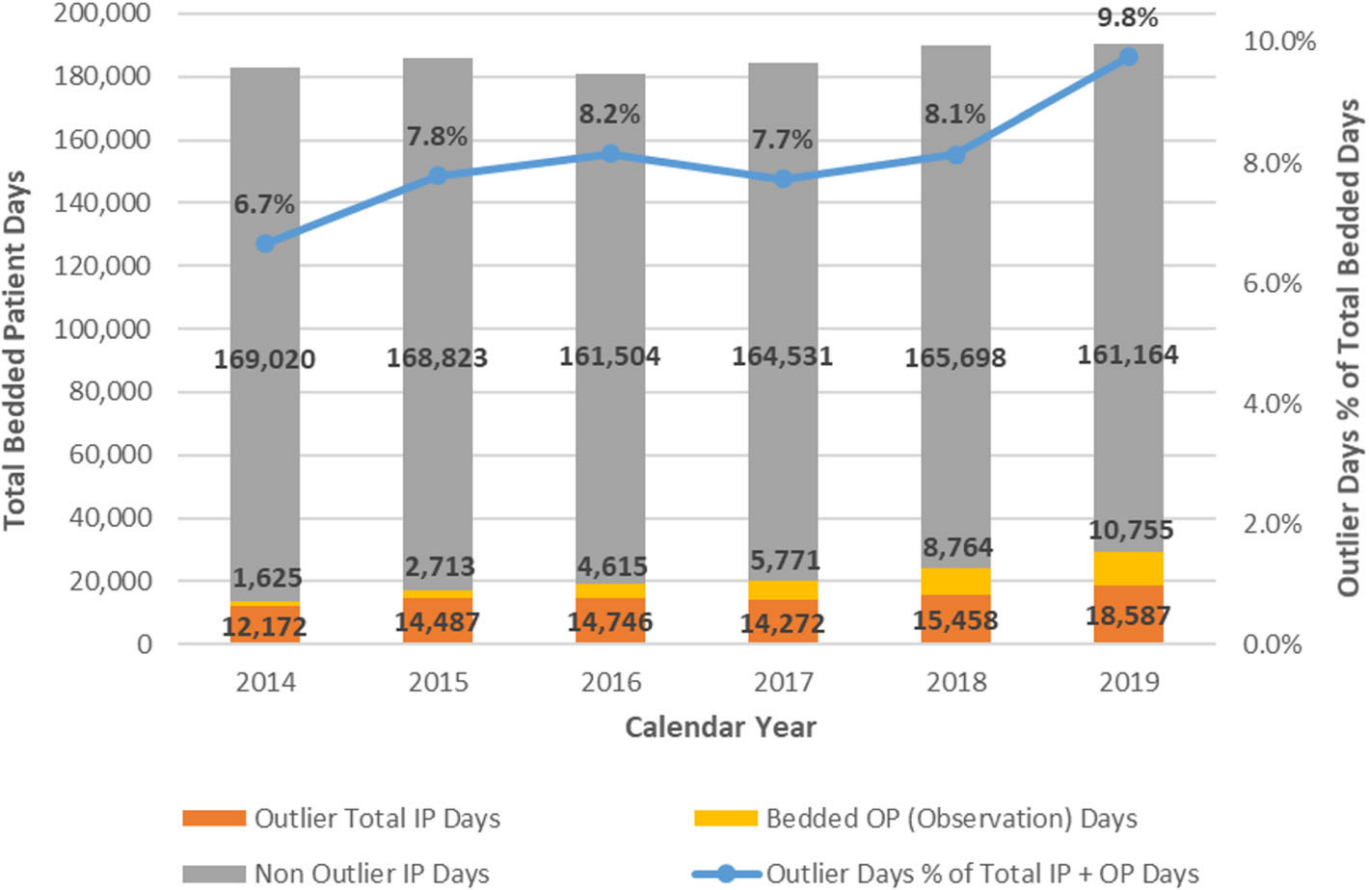
Total Patient Days, Outlier Days Count and % of Total

The total number of outpatient observation days (observation days for patients hospitalized solely in outpatient observation status + any outpatient observation days for those hospitalizations subsequently “converted” to inpatient) increased from 1625 in 2014 to 10,755 in 2019 (Fig. 4).

### Inpatient days and Total Hospital days

The total hospital days (inpatient + outpatient observation days) on the medicine, surgery and neurology services increased during the study period from 182,817 in 2014 to 190,506 in 2019 (Fig. 4). The number of inpatient days (and percentage of total days) attributable to outliers increased from 12,172 (6.7%) in 2014 to 18,587 (9.8%) in 2019. While there was a shift from inpatient towards partial outpatient observation stays, the total number of hospital days attributable to non-outliers was essentially stable from 170,645 in 2014 to 171,919 in 2019 (Fig. 4).

## Discussion

These findings demonstrate that LOS outliers accounted for a significant and disproportionate use of hospital resources which progressively increased from 2014 to 2019. Not only has the total inpatient days accounted for by outliers at our hospital been steadily increasing, the proportion of outlier hospitalizations has also been increasing. Given that Vizient defines outliers as the top 1% LOS by DRG of all admissions nationally, outlier cases as a percent of all admissions went from approximately the national average (0.98%) in 2014 to over 50% more than our peer hospitals in 2019 (1.56%). This represents a nearly 60% relative increase in outlier cases. We also note that the number of inpatient + observation hospital days attributed to non-outliers was essentially stable over the study period, with an increase in total days being attributed mainly to outlier cases. The total number of hospital beds has been static during the study period, with a corresponding increase in bed occupancy to near hospital capacity. This suggests that LOS outlier cases are of concern not only for traditional quality concerns, such as increased adverse patient-care events and excess resource utilization, but also may be “crowding out” non-outlier admissions. This “crowding out” might be expected to result in increased Emergency Department (ED) admission waiting times, and a hospital’s ability to accept new patients. Our concern is supported by data from the Maryland CHATS Region III - County/Hospital Alert Tracking System [18]. This system has a series of capacity alerts, including a hospital’s ED is temporarily overloaded, or the hospital temporarily has no ECG monitored beds available. The number of times JHH was on an alert progressively increased over the study period from 96 times in 2014 to 344 times in 2019 [18]. These serious sequelae of capacity limitations may be driven, at least in part, by the excessive lengths of stay of a relatively few patients.

The dramatic difference in LOS also suggests that outliers are fundamentally different in their hospital course than non-outliers. The source of these excess hospital days is likely attributable to a combination of patient/disease factors, intra-hospital process factors, and extra-hospital factors. Given the stability of non-outlier LOS O/E (Fig. 1), it is unlikely that changes in patient/disease profiles, internal hospital care and/or quality workflows account for increase in outlier days and cases. In line with prior work suggesting that a significant proportion of hospital days are attributable to non-medical delays [17], it is possible, if not probable, that many patients who remain hospitalized at our facility for prolonged periods of time do so because a safe and appropriate discharge plan cannot be formulated. These patients are often absent medical necessity for ongoing acute care hospitalization, with extra-hospital factors predominantly driving the delay. If this is indeed the case, traditional efforts to decrease length of stay that focus on the efficiency of intra-hospital processes would likely be ineffective in the outlier population.

We also note that the total number of observation days increased significantly during the study period. However, “observation” vs. “inpatient” is largely a billing distinction driven by 3rd-party payer definitions. The marked increase likely represents changes in payer-driven status determination rather than significant changes in patient population or care delivery. Finally, the observation days in outlier cases where patients were subsequently “converted” to inpatient were a small component of the total LOS and did not significantly affect the analysis.

Potentially important limitations of this study include that while the sample size was large, the findings represent a single center experience and further study is needed to understand the broader applicability of these findings at other hospitals. Additional studies are also needed to quantify and categorize the factors, both medical and non-medical, that affect the progression of care and hospital discharge for outlier cases.

## Conclusions

This study highlights the outsized and increasing role that LOS outlier cases have on total hospital bed days. These cases utilize a disproportionate share of hospital resources while potentially restricting access to care for other patients due to hospital-bed capacity limitations. This indicates a need to prioritize a comprehensive, targeted approach to address outlier stays to better serve these patients while also improving hospital capacity. This approach should include an analysis of factors external to hospitals, such as local skilled nursing facility capacity, nursing home capacity, and non-hospital based social care services.

## Data Availability

The datasets generated and/or analyzed during the current study are not publicly available due to patient privacy and contractual agreements between Johns Hopkins Hospital and Vizient, Inc. Summary data are available from the corresponding author on reasonable request.
